# Genetic and Molecular Biomarkers in Aggressive Pheochromocytomas and Paragangliomas

**DOI:** 10.3390/ijms25137142

**Published:** 2024-06-28

**Authors:** Francesca Torresan, Clelia Iacobone, Francesco Giorgino, Maurizio Iacobone

**Affiliations:** 1 Endocrine Surgery Unit, Department of Surgery, Oncology and Gastroenterology, University of Padova, 35128 Padova, Italy; francesca.torresan@unipd.it; 2 Department of Precision and Regenerative Medicine and Ionian Area, Section of Internal Medicine, Endocrinology, Andrology and Metabolic Diseases, University of Bari Aldo Moro, 70121 Bari, Italy; c.iacobone2@studenti.uniba.it (C.I.); francesco.giorgino@uniba.it (F.G.)

**Keywords:** pheochromocytomas, paragangliomas, metastases, aggressive tumors

## Abstract

Pheochromocytomas and paragangliomas (PPGLs) are rare neoplasms producing catecholamines that occur as hereditary syndromes in 25–40% of cases. To date, PPGLs are no longer classified as benign and malignant tumors since any lesion could theoretically metastasize, even if it occurs only in a minority of cases (approximately 10–30%). Over the last decades, several attempts were made to develop a scoring system able to predict the risk of aggressive behavior at diagnosis, including the risk of metastases and disease recurrence; unfortunately, none of the available scores is able to accurately predict the risk of aggressive behavior, even including clinical, biochemical, and histopathological features. Thus, life-long follow-up is required in PPGL patients. Some recent studies focusing on genetic and molecular markers (involved in hypoxia regulation, gene transcription, cellular growth, differentiation, signaling pathways, and apoptosis) seem to indicate they are promising prognostic factors, even though their clinical significance needs to be further evaluated. The most involved pathways in PPGLs with aggressive behavior are represented by Krebs cycle alterations caused by succinate dehydrogenase subunits (SDHx), especially when caused by *SDHB* mutations, and by fumarate hydratase mutations that lead to the activation of hypoxia pathways and DNA hypermethylation, suggesting a common pathway in tumorigenesis. Conversely, PPGLs showing mutations in the kinase cascade (cluster 2) tend to display less aggressive behavior. Finally, establishing pathways of tumorigenesis is also fundamental to developing new drugs targeted to specific pathways and improving the survival of patients with metastatic disease. Unfortunately, the rarity of these tumors and the scarce number of cases enrolled in the available studies represents an obstacle to validating the role of molecular markers as reliable predictors of aggressiveness.

## 1. Introduction

Pheochromocytomas are rare tumors that arise from chromaffin tissue of the adrenal medulla, whereas paragangliomas are chromaffin-cell tumors originating from chromaffin cells of extra-adrenal sites along the sympathetic and/or the parasympathetic chain [1]. Paragangliomas are categorized into four groups: aorticosympathetic, branchiomeric, intravagal, and visceral-autonomic. Pheochromocytomas and paragangliomas (PPGLs) have been considered separate groups of tumors until 2022, when the World Health Organization (WHO) proposed changing the nomenclature and classification of these tumors, underlining that pheochromocytomas are neuroendocrine neoplasms that originate from chromaffin cells of the adrenal medulla and can be considered intra-adrenal paragangliomas, and thus recognizing that these two entities are more similar than different [2]. PPGLs are extremely rare tumors, with an incidence of about 2–8 cases per million people per year; they are found in about 4% as incidentalomas [3], but autopsy series have revealed a much higher prevalence [4]. Typically, they can synthesize, store, and secrete catecholamines, with the exception of parasympathetic variants, which are usually non-functioning [1].

Most PPGLs are sporadic, but 25–40% occur in hereditary syndromes such as multiple endocrine neoplasia type 2 (MEN 2), neurofibromatosis type 1, von Hippel–Lindau syndrome, and hereditary PPGLs due to gene mutations in succinate dehydrogenase (*SDHA*, *SDHAF2*, *SDHB*, *C*, *D*), *TMEM-127*, *MAX*, *FH*, *EPAS1/HIF-2α*, or *MDH2* genes [5]. In these cases, they may appear as typically multiple and recurrent localizations, sometimes simulating a metastatic disease. For this reason, PPGLs are considered among the tumors with the highest heredity rate, with a wide range of genes involved; in fact, all PPGL patients should be subjected to genetic screening.

Since 2017, and recently confirmed by the 2022 WHO classification [2], PPGLs are no longer categorized as “benign” and “malignant” but as “metastatic” and “not metastatic” since all PPGLs are considered malignant, although with a variable aggressiveness and tendency to metastasize. Moreover, metastases are defined as the histologically proven presence of PPGL-derived cells in tissues where they are normally absent (i.e., bone and lymph nodes). Thus, the presence of locally recurrent disease after PPGL surgical excision or synchronous or metachronous distant multiple tumors cannot be considered metastases since they can occur as a consequence of local incomplete resection or a misdiagnosed hereditary syndrome, respectively. Most PPGLs are not metastatic; about 10% show metastatic behavior at onset, but this number rises (30%) when considering that metastases have been reported, even after several decades, after initial surgical resection [6,7]. Metastases and recurrences have a significant impact on survival since the 5- and 10-year survival rates for metastatic PPGLs are approximately 65% and 35%, respectively.

Unfortunately, there are currently no clinical, biochemical, histopathological, or molecular markers available to accurately forecast the metastatic behavior of PPGLs at diagnosis. In 2002, Thompson proposed a system based on histopathological features to predict the aggressive course of PPGLs, the PASS score [8]. This system has been validated for pheochromocytomas (and, to a lesser extent, also for paragangliomas), but it seems to be more efficient for predicting the absence of metastatic behavior in cases of lower scores [9]. In 2008, the GAPP score was proposed to predict both overall recurrent disease and distant metastases, including the growth pattern, cellularity, neoplastic necrosis, capsular/vascular infiltration, Ki-67 proliferation index, and type of catecholamine secretion as diagnostic criteria [10]. In 2017, a modified GAPP Score was published, including the results of SDHB immunohistochemistry [10]. In 2019, a new score, the COPPS (Composite pheochromocytoma/paraganglioma Prognostic Score) [11], was also proposed. It includes clinico-pathological features (tumor size, vascular invasion, necrosis) and the losses of S100 and SDHB immunostaining in order to predict the risk of metastases. This score might have a higher specificity and sensitivity than PASS and GAPP, but at the moment, it has not been validated yet. However, all the available scores have an adequate negative predictive value in cases of lower scores but inadequate positive predictive value since higher scores seem to lead to an overestimation of the metastatic risk [2]. The last 2022 WHO classification did not endorse any scoring system; however, it is evident that histopathology alone is not sufficient to predict the long-term outcomes of PPGLs, and it should be integrated with molecular biology analyses.

Over the last 20 years, significant progress has been achieved in the field of molecular biology and in the study of markers related to the aggressive behavior of PPGLs, but their clinical significance has yet to be fully clarified. Currently, the lack of reliable predictors of metastatic disease forces patients with PPGLs to undergo life-long follow-up. Thus, the identification of pathogenetic molecular markers of metastatic behavior in PPGLs is pivotal to identifying cases with negative prognostic outcomes and, eventually, metabolic pathways that can be targeted for new drugs.

Even considering the limitations due to the controversial definition of malignancy in previous literature and the fact that malignant and metastatic diseases have been inappropriately reported as equivalent, the present review summarizes the current findings on the main genetic and potential molecular markers in aggressive PPGLs.

## 2. Genetic Aberrations in PPGLs

Based on transcriptomic studies, PPGLs can be grouped into three main clinically relevant clusters (Table 1).

Cluster 1 (or pseudohypoxia group) includes either germline or somatic germline mutations in succinate dehydrogenase subunits (*SDHx*), fumarate hydratase (*FH*), and the von Hippel–Lindau tumor suppressor (*VHL*) and endothelial PAS domain protein 1 (*EPAS1*) genes; cluster 2 (or kinase signaling group) includes germline or somatic mutations in genes playing a key role in the kinase cascade, such as *Ret* proto-oncogene (*RET*), Neurofibromin 1 (*NF1*), Transmembrane Protein 127 (*TMEM127*), MYC-associated factor X (*MAX*), and *H-Ras* proto-oncogene (*HRAS*); and cluster 3, also known as the Wnt signaling group, consists of recently identified somatic mutations in cold shock domain-containing E1 (*CSDE1*) and somatic gene fusions affecting mastermind-like transcriptional coactivator 3 (*MAML3*) [12,13]. A list of the PPGL susceptibility genes and their related metastatic risk is reported in Table 2.

### 2.1. Cluster 1

**Figure 1:**
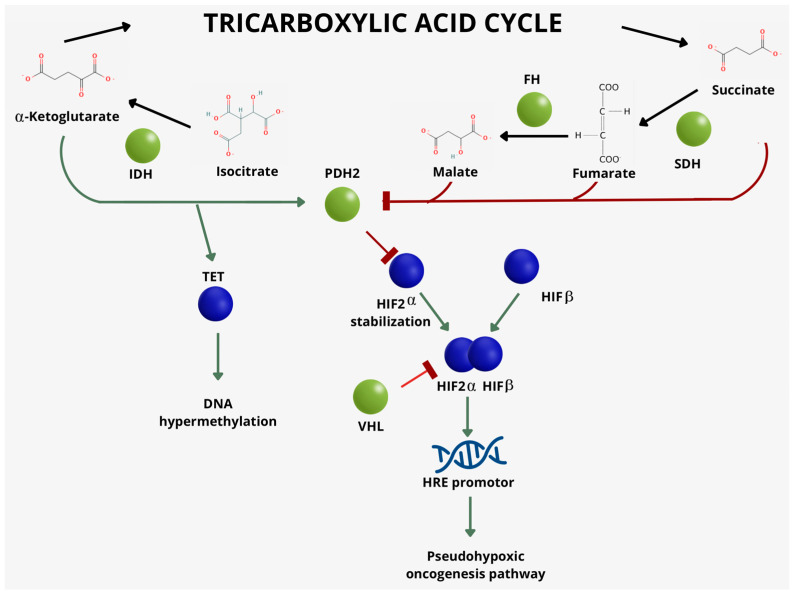
Schematic representation of the main genes and enzymes of the tricarboxylic acid cycle that are involved in cluster 1 pheochromocytomas and paragangliomas.

Cluster 1 PPGLs represent roughly 25–35% of all PPGLs. It comprises either germline or somatic mutations in the *VHL* gene, the *SDHx* gene family (especially *SDHB*, *SDHD* and more recently *SDHAF2*), and other genes involved in the pseudohypoxia response (*HIF2α/EPAS1*, *NOX4*, *LOXL2*) and angiogenesis (*VEGF*) [14]; these genes are normally implicated in the regulation of neoangiogenesis, oxidoreductase activity, and the hypoxia response [15,16]. A further subclassification in cluster 1 has been proposed by other studies: subcluster 1A, which includes PPGLs with mutations of *SDHx* genes and *FH* genes (genes involved in Krebs cycle), and subcluster 1B, which includes mutations in *HIF2α* and *VHL* genes (involved in the pseudohypoxia response) (Figure 1). Even if these subcluster genes are involved in the regulation of distinct cellular functions, when mutated, both of them cause an overactivation of HIF1α and HIF2α, suggesting a common profile of tumorigenesis [14].

*VHL* is a tumor suppressor gene whose mutation is associated with an autosomal dominant disease named von Hippel–Lindau syndrome that results in the development of retinal hemangioblastomas, central nervous system (CNS) hemangioblastomas, PPGLs, and renal carcinomas [17]. The *VHL* gene encodes for a protein, pVHL, that is part of a complex with E3 ubiquitin ligase activity; this complex is involved in the ubiquitination and subsequent degradation of hypoxia-inducible factors (HIFs), which are pivotal transcription factors for controlling gene expression in response to hypoxia [18]. A mutation in the *VHL* gene leads to an imbalance between levels of HIF1α (which is a pro-apoptotic factor) and HIF2α. HIF2α interacts with cyclin D1; hyperactivation of cyclin D1 results in higher proliferation and decreased rates of apoptosis [18,19]. Recent studies show that VHL is also implied in cellular differentiation, adhesion of cells to cells and cells to matrix, and apoptosis [20]. Mutations in the *VHL* gene were also found to be associated with increased levels of HIF1α target genes, genes involved in apoptosis (EGLn3), and metastasis [21,22,23]. The activation of these pathways might suggest that VHL-related PPGLs could be associated with predominantly aggressive behavior, but this is not the case. In fact, VHL-related PPGLs have a low rate of metastatic potential; patients with familial pheochromocytomas in VHL syndrome were found to have metastatic or locally aggressive tumors in 8% of cases [24].

The SDH enzyme complex includes four nuclear-encoded subunits (SDHA, SDHB, SDHC, SDHD). Two other factors are involved in assembling the complex (SDHAF1, SDHAF2). This enzyme complex converts succinate to fumarate, which releases electrons as part of the citric acid cycle, and the enzyme complex additionally provides an attachment site for released electrons to be transferred to the oxidative phosphorylation pathway. The SDH enzyme complex plays a role in oxygen-related gene regulation through its conversion of succinate, which is an oxygen sensor that stabilizes the HIF1 transcription factor [25]. Mutations in the SDHx complex lead to an increased level of succinate, which inhibits PHD enzymes, leading to the accumulation of HIFα [15].

Mutations in *SDH* genes are the most frequent in cluster 1, representing almost half of the cases, and cause the well-known paraganglioma-pheochromocytoma syndromes (PPGL 1, 2, 3, 4, and 5 syndromes) (Table 3) [26].

SDHD-related PPGLs (PPGL 1 syndrome) in the head and neck have a parasympathetic origin and are mostly nonfunctional. Although *SDHD* mutation carriers may develop multiple head and neck PPGLs, metastatic disease is not frequent (15–29%) [27,28].

In PPGL 2, associated with *SDHAF2* mutations, no cases of metastatic PPGLs have been recorded [29].

Mutations of the *SDHC* gene (PPGL 3 syndrome) are often associated with head and neck PPGLs with limited metastatic behavior [30].

Mutations in *SDHA* (PPGL 5 syndrome) are extremely rare when compared to other *SDHx* and, consequently, less characterized. Anyways, recent studies illustrate that *SDHA*-related PPGLs are highly associated with metastases (30–66%) [31,32].

Mutations in *SDHB* in PPGLs are proven to strongly predict the aggressive and metastatic behavior in PPGL 4 syndrome [33]. *SDHB*-mutated tumors are characterized by a hypermethylation phenotype as a result of the accumulation of the oncometabolites succinate and fumarate. In particular, *SDHB*-mutated PPGLs were shown to acquire a hypermethylation phenotype through the inhibition of TET (an enzyme involved in the DNA demethylation process). Mutations in the *TET* gene, in vitro, led PPGL cells to acquire invasive behavior and—when associated with HIF2α mutations—to the development of metastatic behavior [34]. In fact, *SDHB*-mutated tumors are reported to be those with the highest risk of metastasis (66–83%) [35]. Moreover, the promoter methylation of several other genes (i.e., *p16INK4A*, *RASSF1A*, *NORE1A*, *p16INK4A*, *RARB*, *DCR2*, *CDH1*, and *APC*) has been shown to influence tumor aggressiveness [36,37,38].

Fumarate hydratase (FH) is an enzyme that converts fumarate to malate. The inactivation/deficiency of FH causes an intracellular accumulation of fumarate, which is structurally resemblant to succinate, which is an oxygen sensor that stabilizes the HIF1 transcription factor. FH mutations in PPGLs are rare; a study conducted on 598 samples of PPGLs showed that mutations in FH were found in 0.83% of cases, but aggressive behavior was found in 60% of those cases [39].

HIF2α is a factor involved in the physiological response to oxygen levels. It is also essential for the preservation of catecholamine homeostasis and the prevention of heart failure during the early phases of embryonic development. The HIF complex is involved in several fundamental functions related to chromaffin cells, namely angiogenesis, glucose metabolism, migration and invasion, proliferation, and differentiation through transcription of hypoxia-inducible genes; HIF2α promotes the proliferation of human placenta-derived mesenchymal stem cells through the MAPK/ERK signaling pathway [18]. hPMSCs over-expressing HIF-2α showed higher proliferative potential and higher expression of CCND1 (CyclinD1), MYC (c-Myc), POU5F1 (Oct4), and the components of the MAPK/ERK pathway [40]. It is common knowledge that HIF-1α is actively involved in tumorigenesis, whereas HIF-2α is predominant in metastases and chemoresistance in different solid tumors (such as renal carcinoma, non-small cell lung carcinomas, and glioblastoma) [41]. Somatic mutations in HIF2α have been discovered in sporadic PPGLs, suggesting that HIF2α has a role in the development of aggressive behavior [42], with an estimated risk of developing metastasis of about 30% [18].

Other mutations of genes involved in the Krebs cycle and mitochondrial metabolism have been recently found in metastatic PPGLs, including *SLC25A11* [43], *GOT 2* [44], and *DLST* [45].

The *SLC25A11* gene encodes the mitochondrial 2-oxoglutarate/malate carrier, and germline mutations have been identified in metastatic PPGLs. In fact, *SLC25A11* germline mutations have been recently reported in PPGLs with an SDH-like molecular profile without *SDHx* or *FH* mutations. In most of these patients with germline *SLC25A11* mutations, a loss of heterozygosity was also reported, thus suggesting a role of *SLC25A11* as a tumor-suppressor gene [46]. In fact, in a cohort of 121 patients with metastatic PPGLs, 5% of all metastatic patients were *SLC25A11* mutation carriers; a malignant phenotype was observed in five out of the seven (71%) of them [46], suggesting that *SLC25A11* can be considered a novel PPGL susceptibility gene and that loss of function is correlated with metastatic behavior.

In recent years, other molecular markers have been discovered and correlated with a high metastatic potential, including the *TERT* promoter, *ATRX* mutations, and the *MAML3* fusions gene [47]. HTERT mRNA was expressed in both aggressive and not aggressive PPGLs, but its levels of expression were higher in aggressive tumors [48]. Also, heat shock protein 90 (HSP90) levels were found to be increased in aggressive PPGLs [49]; cyclo-oxygenase [50], N-cadherin [51], VEGF, ET-A, and ET-B34 were also found overexpressed in aggressive PPGLs, suggesting that these could be markers used to predict metastatic behavior. Recently, lower levels of EM66 were found in the so-called malignant tissue compared to benign [52]. The gamma-tubulin, *GCSF2*, and *IL-2R* genes were found to be aberrantly expressed in aggressive PPGLs [53]. However, further studies are needed to confirm the role of these genetic markers.

### 2.2. Cluster 2

**Figure 2:**
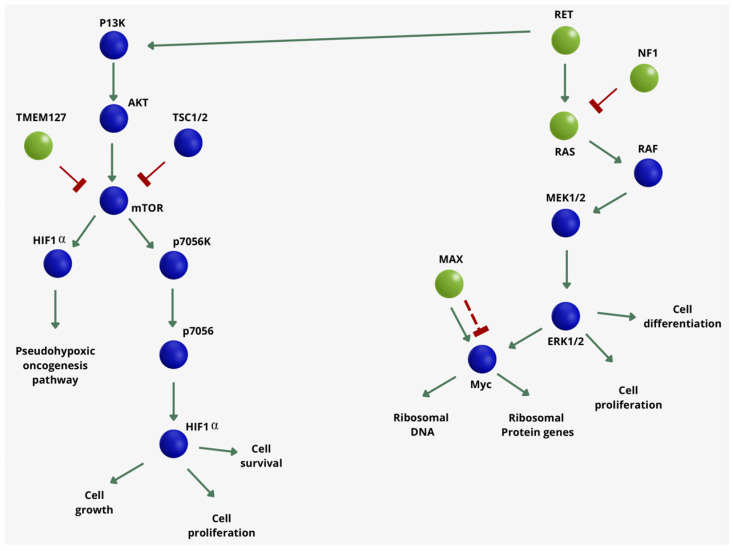
Schematic representation of the main genes involved in cluster 2 pheochromocytomas and paragangliomas.

Cluster 2 (kinase signaling pathway) (Table 1) includes germline or somatic mutations in the rearranged-during-transfection (*RET*) proto-oncogene, neurofibromin 1 (*NF1*) tumor suppressor, transmembrane protein 127 (*TMEM127*), Myc-associated factor X (*MAX*), and somatic mutation in *H-RAS*. Mutations in this cluster of genes activate the uncontrolled cell proliferation pathway (Figure 2). It is known that the risk of metastatic disease in cluster 2 is low: the predisposition to an aggressive course is <3% for *RET* mutations and around 11% for *NF1* mutations [54].

*RET* is a proto-oncogene encoding for a receptor tyrosine kinase that acts as a receptor for proteins of the glial cell line-derived neurotrophic factor (GDNF) family and is also involved in extracellular signaling pathways. It is also involved in the development of the sympathetic, parasympathetic, and enteric nervous systems [55]. Gain-of-function germline mutations are associated with the development of MEN type 2A and 2B [56] syndromes, which cause tumors in the thyroid and parathyroid glands and adrenal glands that are characterized by mutations on the extracellular and intracellular domain of the RET receptor, respectively, that subsequently dimerize even in the absence of ligand, leading to overexpression of the oncogene [57]. Aggressive and metastatic pheochromocytoma are rare in MEN2 syndrome (<5%), even though MEN2B represents the more aggressive variant [13].

*NF1* is a gene encoding for neurofibromin 1, a GTPase-activating protein that negatively regulates RAS/MAPK pathway activity by accelerating the hydrolysis of Ras-bound GTP [58]. Germline mutations of *NF1* are associated with an autosomal dominant disorder called neurofibromatosis type 1. The most common signs of this disease are café-au-lait spots, skin and plexiform neurofibromas, Lisch nodules, bone defects, optic nerve gliomas, life-threatening malignant peripheral nerve sheath tumors, attention deficits, learning deficits, and other cognitive disabilities. Other rare manifestations of the disease can be gastrointestinal stromal tumors, astrocytic neoplasms, juvenile myelomonocytic leukemias, Watson syndrome, pheochromocytomas, and breast cancer [59]. The risk of developing pheochromocytomas in NF1 patients is low (0.1–5.7%) [60], but an aggressive course has been reported in 12% of cases [13].

*TMEM 127* is a tumor suppressor gene encoding the transmembrane protein 127, which is a negative regulator of mTOR. Loss of function mutations in *TMEM127* are notably associated with pheochromocytoma. Normally, *TMEM 127* suppresses tumor development by promoting RET ubiquitination and degradation. TMEM127 is a component of the ubiquitin system; its mutation causes RET stabilization, and this mechanism was discovered to be implicated in the tumorigenesis of pheochromocytoma [61]. Most patients with *TMEM127* germline mutations present with apparently benign-course (98%), unilateral (approximately 70%) tumors that are almost exclusively adrenal (96%). The presence of metastases has been detected in a few cases [62].

The *MAX* gene encodes for a protein member of the basic helix-loop-helix leucine zipper (bHLHZ) family of transcription factors [63]. Together with Mad, Mxi1, and Myc, it is able to form both homodimers and heterodimers. Myc is an oncoprotein implicated in cell proliferation, differentiation, and apoptosis. The homodimers and heterodimers compete for a common DNA target site (the E box), and rearrangement among these dimer forms provides a complex system of transcriptional regulation. Hereditary PPGLs have been linked to mutations in this gene. The *MAX* gene was discovered as a susceptibility gene in 2012 when a cohort of 1694 patients with PPGLs was analyzed [64]. *MAX* germline mutations are responsible for the disease in 1.12% of cases. *MAX* somatic mutations have been found in 1.65% of PPGLs. Patients with *MAX* mutations developed metastatic disease in around 10% of cases [13].

The *KIF1B* gene encodes for a protein called kinesin family member 1B, part of the kinesin family of proteins [65]. These proteins are essential for the transport of materials within cells. Kinesin proteins function like freight trains that transport cargo, and their structure is suited for this cargo-carrying function. One part of the protein, called the motor domain, provides the power to move the protein and its cargo along a track-like system made from structures called microtubules. Another part of the kinesin protein, which varies among members of this protein family, binds to specific materials for transport. In addition to transport functions, the kinesin family member 1B protein is involved in programmed cell death (apoptosis). *KIF1B* gene mutations have been reported in individuals with pheochromocytoma [13,66,67]. Mutations in the *KIF1B* gene also lead to the development of neuroblastoma and Charcot–Marie–Tooth disease.

RAS is a family of proteins with GTPase activity involved in cell growth, differentiation, and survival. Mutations in *RAS* genes can cause hyperactivation of RAS signaling, resulting in the activation of the RAF/ERK and PI3K/AKT/mTOR signaling pathways—even in the absence of incoming signals—and leading to cancer. The three *RAS* genes in humans (*H-RAS*, *K-RAS*, and *N-RAS*) are the most common oncogenes in human cancer; mutations that permanently activate Ras are found in 20 to 25% of all human tumors and in up to 90% in certain types of cancer [68].

Recently, somatic *H-RAS* mutations in sporadic PPGLs were identified by exome sequencing, showing a frequency of 6.9% in a cohort of 58 tumors analyzed. These tumors displayed activation of the RAS/RAF/ERK signaling pathway and were associated with male predominance and clinically benign behavior [69]. A study performed in 2014 showed the presence of the *H-RAS* mutation in 5.2% of cases, which were confined to sporadic PPGLs; *H-RAS* mutations lacked any significant correlation with pathological or basic clinical endpoints [70].

### 2.3. Cluster 3

Cluster 3 is a purely somatic cluster that involves the Wnt signaling pathway and includes mutations in only two genes, *MAML3* and *CSDE1*. MAML3, also known as mastermind-like transcriptional coactivator 3, is a transcription coactivator involved in the Notch signaling pathway. Elevated β-catenin protein expression and elevated mRNA expression of WNT4 were found in tumors with *UBTF-MAML3* gene fusions. Moreover, these gene fusions led to protein gain of function and DNA hypomethylation and were found to be associated with aggressive PPGLs [16].

### 2.4. Other Genes Involved in PPGL Oncogenesis

Several studies analyzed the role of *p53* in the tumorigenesis of sporadic PPGLs. *P53* is a tumor suppressor whose mutations are frequently found in different types of malignant tumors. p53 is a protein involved in guarding the stability of the genome. In case of DNA damage, it downregulates the cell cycle and inhibits cell division, causing apoptosis [71]. Germline mutations in *p53* are associated with Li Fraumeni syndrome, a rare familial syndrome with a high incidence of diverse cancers, including adrenocortical carcinoma. Yoshimoto described the relatively high incidence of *p53* gene mutations or intronic sequence alteration in multiple and aggressive PPGLs but not in benign solitary cases, suggesting that *p53* mutation could play some role in the pathogenesis of multiple and/or aggressive PPGLs [72].

Several other genes involved in PPGL tumorigenesis are emerging after the advent of next-generation techniques and whole-genome sequencing, such as *ATRX*, *CDKN2A/p16*, and *GNAS*, but their role is still debated.

## 3. Molecular and Biochemical Markers

Besides genetic aberrations, several potential molecular markers have been investigated to differentiate between PPGLs with benign and aggressive courses.

According to the European Society of Endocrinology Clinical Practice Guidelines [73], the measurement of catecholamines and their metabolites metanephrines (normetanephrine and metanephrine) and 3-methoxytyramine is strongly recommended in metastatic PPGLs; in fact, an increase in their levels after the complete resection of the primary tumor suggests the progression of disease (metastasis or recurrence) or new tumor development. Higher plasma and 24 h urinary levels of metanephrines have been reported in metastatic PPGLs, as described by Feng et al. [74]. The mean catecholamine concentration was also significantly higher in malignant than in benign tumors. However, in another study including Indian patients, significantly lower plasma metanephrine levels were found in metastatic compared to benign PPGLs [75].

Higher levels of plasma 3-methoxytyramine were detected in metastatic PPGLs, mainly in SDHB-mutated PPGLs [76,77]. Moreover, dopamine- and 3-methoxytyramine (the O-methylated dopamine metabolite)-secreting PPGLs were found to be associated with a higher risk of metastatic potential and a worse tumor prognosis [78,79]. Generally, most PPGLs related to cluster 1 are characterized by a noradrenergic biochemical phenotype producing noradrenaline and/or dopamine that can explain higher levels of normetanephrine and 3-methoxytyramine compared with metanephrine in metastasizing PPGLs [80].

A recent study by Murakami M et al. [81] reporting tissue metabolome analysis using MALDI–mass spectrometry in a large cohort of PPGLs revealed specific metabolic alterations related to genetic abnormalities of cluster 1 and 2 PPGLs. In particular, lower levels of kynurenine metabolites derived from tryptophan catabolism, such as xanthurenic acid, were found in cluster 1 tumors compared to cluster 2. Lower levels of xanthurenic acid were associated with shorter survival, and it was considered an independent risk factor for metastatic diseases. It has been hypothesized that kynurenine pathway metabolites may have antiproliferative functions like in colon and renal cell cancers.

The measurement of Krebs cycle metabolites, in particular the tissue succinate-to-fumarate ratio, by liquid chromatography–mass spectrometry has been used to assess the functionality of SDH, mainly when genetic variants of unknown significance are present since it is unknown how the mutation affects function. In fact, despite immunohistochemistry being the preferred method for assessing SDH function, unfortunately, it does not evaluate functionality at the enzymatic level. An innovative approach is to measure the metabolite precursors and products of enzymatic processes using liquid chromatography–mass spectrometry. A recent study by Wallace PW et al. [82] revealed that metabolite profiling has higher diagnostic specificity compared to SDHB immunohistochemistry (99.2% vs. 92.5%, *p* = 0.021). In this study, a machine learning approach was also applied, revealing that SDHB immunohistochemistry and metabolite profiling are complementary tools for evaluating SDH status in PPGL tissues.

miRNAs are another class of potential diagnostic and prognostic molecular markers. A recent study reporting a comprehensive analysis of miRNomes from 443 PPGLs identified six miRNAs (miR-21-3p, miR-183-5p, miR-182-5p, miR-96-5p, miR-551b-3p, and miR-202-5p) associated with metastatic potential [83]. In particular, the co-expression of miR-21-3p/miR-183-5p was found to accurately predict a metastatic potential in PPGLs. It was reported that miR-21-3p interferes in the regulation of mTOR pathways through the downregulation of TSC2 mRNA. Under normal conditions, the TSC2 complex ubiquitinates and consequently degrades mTOR. Therefore, the downregulation of TSC2 mRNA may lead to the hyperactivation of the mTOR/AKT pathway. Finally, it was demonstrated in vitro that miR-21-3p overexpression enhanced the sensitivity of the cells to rapamycin, thus indicating miR-21-3p as a possible future marker to select patients that may benefit from mTOR inhibitors.

## 4. Conclusions

PPGLs are rare tumors with uncertain behavior and potentially metastatic. To date, several genetic, molecular, and biochemical markers predicting aggressive behavior have been proposed. However, the majority remain controversial, and the prediction of prognosis is challenging. Moreover, most of the published works have severe limitations, including small sample size, the rarity of metastasizing cases, tumor sets with a predominance of pheochromocytomas, and a lack of follow-up. The same definition of malignancy is controversial, and it has been clarified only in recent years [2].

Thus, although many features have been suggested as potential predictive factors of aggressiveness, the majority of them remain controversial.

Nevertheless, a combination of different genetic and molecular markers might predict an aggressive potential in PPGLs, thus allowing stricter and more intensive follow-up in high-risk patients. Moreover, establishing the pathways of tumorigenesis is fundamental to developing new-generation drugs with specific target pathways in order to improve the survival of patients with metastatic disease.

## Figures and Tables

**Table 1:** Cluster classification of the known susceptibility genes.

		Germline and Somatic Mutations	Exclusively Somatic Mutations
**Cluster 1** (Pseudohypoxic cluster)	Cluster 1A	*SDHx (A/B/C/D)*, *SDHAF2*, *FH*, *MDH2*, *GOT2*, *DLST*, *SLC25A11*	*IDH1/2*
	Cluster 1B	*EGLN1/2*, *VHL*	*EPAS1 (HIF2α)*
**Cluster 2** (Kinase signaling cluster)		*RET*, *MERTK*, *MET*, *NF1*, *MAX*, *TMEM127*	*FGFR1*, *HRAS*, *BRAF*
**Cluster 3** (Wnt signaling cluster)		-	*CSDE1*, *UBTF-MAML3*

**Table 2:** PPGL susceptibility genes and the associated risk of aggressive behavior.

Gene	Function	Risk of Aggressive Behavior
***VHL***—von Hippel–Lindau	Regulates HIF degradation	Low (8%)
***SDHA***—Succinate dehydrogenase subunit A	Subunit of mitochondrial protein complex SDH	High (30–66%)
***SDHB***—Succinate dehydrogenase subunit B	Subunit of mitochondrial protein complex SDH	High (66–83%)
***SDHC***—Succinate dehydrogenase subunit C	Subunit of mitochondrial protein complex SDH	Low
***SDHD***—Succinate dehydrogenase subunit D	Subunit of mitochondrial protein complex SDH	Medium (15–29%)
***SDHAF2***—Succinate dehydrogenase complex assembly factor 1	Participates in assembly of mitochondrial protein complex SDH	Low
***FH***—Fumarate hydratase	Catalyzes the conversion of fumarate to malate	High (60%)
***RET***—Rearranged during transfection	Tyrosine kinase receptor	Low (<5%)
***NF1***—Neurofibromin 1	Inhibits RAS signaling pathway	Medium-High (12%)
***TMEM127***—Transmembrane protein 127	Inhibits mTOR signaling pathway	Low (<5%)
***MAX***—MYC-associated factor X	Participates in cellular proliferation, differentiation, and apoptosis	Medium (10%)
***Tp53***—Tumor suppressor protein 53	Regulates cell cycle	High

**Table 3:** Genotype–phenotype correlation in pheochromocytoma/paragangliomas syndrome.

Syndrome	Gene, Chromosome Location	Metastatic Disease (%)	Recurrence (%)	Associated Tumors
PPGL1	*SDHD*, *11q23.1*	15–29	60	Gastrointestinal stromal tumors, pituitary tumors
PPGL2	*SDHAF2*, *11q12.2*	-	unknown	
PPGL3	*SDHC*, *1q23.3*	8–28	unknown	Renal carcinoma, gastrointestinal stromal tumors
PPGL4	*SDHB*, *1p36.13*	66–83	unknown	Renal carcinoma, gastrointestinal stromal tumors, pituitary tumors
PPGL5	*SDHA*, *5p15.33*	30–66	unknown	Gastrointestinal stromal tumors, pituitary tumors

## Data Availability

Not applicable.
